# Disease-context dependence of periventricular diffusivity and DTI-ALPS in asymptomatic moyamoya disease

**DOI:** 10.3389/fneur.2026.1927018

**Published:** 2026-10-21

**Authors:** Haitao Liu, Xingchen Liu, Hang Ji, Anqi Xiao, Yi Liu

**Affiliations:** 1 Department of Neurosurgery, West China Hospital, Sichuan University, Chengdu, China; 2 Department of Otolaryngology Head and Neck Surgery, West China Hospital, Sichuan University, Chengdu, China

**Keywords:** chronic cerebral hypoperfusion, cognitive impairment, diffusion MRI, DTI-ALPS, glymphatic system, moyamoya disease, periventricular diffusivity, vascular cognitive impairment

## Abstract

Asymptomatic moyamoya disease, chronic cerebral hypoperfusion, periventricular diffusivity (PVeD), and diffusion tensor image analysis along the perivascular space (DTI-ALPS) are relevant to the study of brain fluid transport and vascular cognitive impairment. PVeD has recently been proposed as a diffusion MRI marker related to amyloid burden and cognition in the Alzheimer's disease continuum, but whether this marker has similar cognitive relevance in chronic hypoperfusion remains unclear. We studied 59 patients with asymptomatic moyamoya disease who had complete demographic, cognitive, diffusion MRI-derived imaging, white matter hyperintensity (WMH), and bilateral Suzuki staging data. Age, sex, MoCA, MMSE, bilateral and mean ALPS indices, and bilateral and mean PVeD indices were analyzed using Spearman correlation, age- and sex-adjusted partial correlation, and multivariable linear regression. False discovery rate (FDR) correction was applied to the correlation analyses. After age- and sex adjustment and FDR correction, *five* of the *six* ALPS-cognition partial correlations remained significant (mean, left, and right ALPS with MoCA; mean and left ALPS with MMSE), whereas *non*e of the *six* PVeD-cognition partial correlations survived FDR correction. In sensitivity models additionally adjusted for WMH burden and maximum hemispheric Suzuki stage, mean ALPS remained associated with MoCA, whereas its association with MMSE was attenuated; mean PVeD was not associated with either cognitive score. These cross-sectional findings suggest that the cognitive relevance of diffusion-based brain fluid markers may vary according to the underlying disease context, structural white matter injury, and angiographic disease severity, and support further longitudinal validation in vascular populations.

## Introduction

Brain fluid transport has become an increasingly important topic in studies of cognitive impairment, particularly across the Alzheimer's disease (AD) continuum and related neurological disorders. The glymphatic hypothesis proposes that cerebrospinal fluid enters the brain along perivascular pathways, exchanges with interstitial fluid, and contributes to clearance of interstitial solutes, including amyloid-beta (1). Impaired cerebrospinal fluid-interstitial fluid exchange has been linked to amyloid deposition, tau-related pathology, neuroinflammation, and cognitive decline in AD (2). Because direct assessment of these processes is difficult in routine clinical studies, diffusion MRI-derived markers have been explored as indirect, non-invasive measures of brain fluid transport.

Diffusion tensor image analysis along the perivascular space (DTI-ALPS) is one of the most widely used MRI-based approaches in this field. The ALPS index was originally proposed to estimate diffusivity along medullary perivascular spaces and was reported to correlate with Mini-Mental State Examination scores in patients with AD (3). Its clinical appeal lies in its non-invasive nature, simple post-processing requirements, and applicability to existing diffusion MRI datasets. However, ALPS is not a direct measure of glymphatic flow. It may be affected by region-of-interest placement, white matter fiber architecture, crossing fibers, scanner parameters, and disease-related tissue injury (4, 5).

More recently, Chen et al. (6) proposed periventricular diffusivity (PVeD) as another diffusion MRI marker in the AD continuum. In that study, lower PVeD was associated with greater amyloid burden, neurodegeneration, cognitive impairment, and clinical severity, with evidence of modification by APOE epsilon4 status (6). These findings suggest that periventricular diffusion signals may be sensitive to AD-related alterations in brain fluid transport or periventricular tissue microstructure.

Whether this interpretation applies to chronic ischemic conditions remains uncertain. Chronic cerebral hypoperfusion is an important vascular contributor to cognitive impairment and may interact with AD-related neurodegenerative processes (7). Moyamoya disease provides a clinically relevant model for studying chronic cerebral hypoperfusion because it is characterized by progressive intracranial arterial stenosis, long-standing hemodynamic compromise, and variable cognitive dysfunction (8). In asymptomatic moyamoya disease, diffusion-derived markers can be examined in a setting with relatively limited interference from clinically overt infarction or intracranial hemorrhage. However, chronic hypoperfusion, white matter injury, periventricular tissue vulnerability, and vascular cognitive impairment may all influence periventricular diffusion signals (8, 9). Therefore, the performance of PVeD observed in the AD continuum should not be assumed to be identical in chronic ischemic populations.

In this Brief Research Report, we compared the associations of PVeD and DTI-ALPS with global cognitive performance, assessed using the Montreal Cognitive Assessment (MoCA) and Mini-Mental State Examination (MMSE), in patients with asymptomatic moyamoya disease. We hypothesized that the cognitive relevance of PVeD may depend on disease context and may not necessarily exceed that of DTI-ALPS in chronic cerebral hypoperfusion.

## Materials and methods

### Study design and participants

This cross-sectional observational study included 59 patients with asymptomatic moyamoya disease who had complete demographic, cognitive, and diffusion MRI-derived imaging data. Moyamoya disease was diagnosed by stenosis or occlusion of the terminal internal carotid artery and/or proximal anterior or middle cerebral arteries, with abnormal basal collateral vessels on vascular imaging. Asymptomatic moyamoya disease was defined as the absence of clinically overt ischemic stroke or intracranial hemorrhage before enrollment. This cohort was selected as a clinical model of chronic cerebral hypoperfusion with limited interference from overt infarction or hemorrhagic injury.

Eligible patients had available data on age, sex, MoCA, MMSE, DTI-ALPS, and PVeD. Patients were excluded for incomplete demographic, cognitive, or imaging data, poor-quality diffusion MRI, or structural brain lesions that precluded reliable ALPS or PVeD measurement. The study was approved by the Biomedical Research Ethics Committee of West China Hospital, Sichuan University. The requirement for written informed consent was waived because of the retrospective observational design and use of de-identified clinical and imaging data.

Angiographic severity was characterized using left and right Suzuki staging recorded in the study dataset. Hemispheric stages were analyzed separately for descriptive laterality assessment. For analytic coding only, an angiographically uninvolved hemisphere was coded as 0; this code does not represent a conventional Suzuki stage. Affected hemispheres in this cohort were classified as Suzuki stages 1–4. To provide a parsimonious patient-level measure of angiographic severity for multivariable sensitivity analyses and to avoid entering two correlated hemispheric stage variables in a cohort of 59 participants, the maximum hemispheric Suzuki stage across the two sides was used as the patient-level covariate. Unilateral involvement was defined as analytic code 0 on one side with Suzuki stage >0 on the contralateral side; bilateral involvement was defined as Suzuki stage >0 on both sides.

### Cognitive assessment

Global cognitive performance was assessed using the MoCA and MMSE. Both scores were analyzed as continuous variables, with higher scores indicating better performance. These instruments were treated as global cognitive screening measures rather than domain-specific neuropsychological tests.

### MRI acquisition

MRI was performed using a 3.0-T scanner (MAGNETOM Prisma; Siemens Healthineers, Erlangen, Germany; syngo MR XA30) with a 64-channel head/neck coil. The protocol included three-dimensional T1-weighted imaging, axial T2-weighted imaging, fluid-attenuated inversion recovery (FLAIR), susceptibility-weighted imaging (SWI), and diffusion tensor imaging (DTI). For three-dimensional T1-weighted imaging, the repetition time/echo time/inversion time was 1,630/2.32/942 ms, the flip angle was 8°, and the slice thickness was 1 mm. For T2-weighted imaging, the repetition time/echo time was 4,530/95 ms, the flip angle was 120°, and the slice thickness was 5.5 mm. For FLAIR, the repetition time/echo time/inversion time was 5,500/98/1,920 ms, the flip angle was 120°, and the slice thickness was 5.5 mm. SWI was acquired with a repetition time/echo time of 27/20 ms, a flip angle of 20°, and a voxel size of 0.45 × 0.45 × 2.50 mm^3^. DTI was acquired using two-dimensional echo-planar imaging with a repetition time/echo time of 3,900/85 ms, a flip angle of 90°, an isotropic voxel size of 2 × 2 × 2 mm^3^, and a multiband acceleration factor of 4. The diffusion scheme included seven b = 0 images and 36, 36, and 49 diffusion-weighted directions at b = 1,000, 2,000, and 3,000 s/mm^2^, respectively. All MRI data underwent quality control before quantitative analysis; images with substantial head motion, signal loss, geometric distortion, severe artifact, or registration failure were excluded from the corresponding analysis.

### DTI preprocessing

DTI data were preprocessed using the FMRIB Software Library (FSL, version 6.0.7.6; FMRIB Analysis Group, University of Oxford, Oxford, UK). Reverse phase-encoded non-diffusion-weighted images were first used to estimate and correct susceptibility-related geometric distortion. TOPUP and EDDY were then applied to correct susceptibility distortion, eddy-current effects, and participant motion, with concurrent rotation of the diffusion-gradient directions. Brain extraction was performed on the corrected mean *b* = 0 image to generate a brain mask. For diffusion-tensor fitting and derivation of fractional anisotropy, mean diffusivity, and directional diffusivity maps (Dxx, Dyy, and Dzz), only the *b* = 0 and *b* = 1,000 s/mm^2^ data were used in DTIFIT. The resulting tensor-derived maps were registered to MNI152 standard space for subsequent DTI-ALPS ROI placement and parameter extraction. Registered images and ROIs were visually inspected in each participant, with manual correction of alignment when necessary to ensure appropriate anatomical correspondence. All correction outputs, brain masks, and spatial registrations underwent visual quality control; images with substantial motion artifact, signal loss, failed distortion correction, incomplete brain extraction, or unacceptable spatial registration were excluded.

### DTI-ALPS index

The DTI-ALPS index was calculated according to previously described methods (3, 5). Predefined fixed regions of interest (ROIs) in MNI152 standard space were used bilaterally at the level of the bodies of the lateral ventricles within projection-fiber and association-fiber regions. The same ROI templates were applied to all participants after spatial registration. Each participant's registered ROIs were visually inspected and, when necessary, manually adjusted to confirm appropriate anatomical localization within the intended projection- and association-fiber regions and to ensure that other major fiber bundles were not included. At this level, deep medullary veins and their surrounding perivascular spaces run predominantly along the left-right direction, corresponding to the x axis; projection fibers run predominantly along the z axis; and association fibers run predominantly along the y axis. The left and right ALPS indices were calculated as ALPS index = mean(Dxx,proj, Dxx,assoc) / mean(Dyy,proj, Dzz,assoc), where Dxx,proj and Dxx,assoc denote diffusivity along the x axis in the projection-fiber and association-fiber ROIs, respectively; Dyy,proj denotes diffusivity along the y axis in the projection-fiber ROI; and Dzz,assoc denotes diffusivity along the z axis in the association-fiber ROI. The mean ALPS index was calculated as the arithmetic mean of the left and right ALPS indices. Because ROI definitions were template based rather than freely drawn on a subject-by-subject basis, conventional inter-rater reliability for ROI placement was not directly applicable; manual review served as quality control for registration and anatomical localization.

### Periventricular diffusivity

PVeD was calculated from each participant's DTI data using the publicly available EstPVeD automated pipeline in MATLAB R2024a and SPM12, following the previously described approach (6). The pipeline uses the mean diffusivity map and Dxx, Dyy, and Dzz tensor components, registers a predefined lateral-ventricular template to diffusion space, and constructs a periventricular analysis region using anatomically adaptive dilation. This region is constrained by tissue-segmentation results to exclude cerebrospinal fluid and non-brain tissue and thereby reduce partial-volume effects. The transverse tensor ratio (TTR) was calculated voxelwise within the final periventricular white-matter region as TTR = Dxx / sqrt(Dxx^2^ + Dyy^2^ + Dzz^2^). The median TTR values within the left and right periventricular regions were extracted as the left and right PVeD measures, respectively, and mean PVeD was calculated as their arithmetic mean. All lateral-ventricle registrations, tissue segmentations, and periventricular masks underwent visual quality control. The EstPVeD workflow generates subject-specific masks automatically rather than through manual ROI delineation. PVeD was interpreted as an indirect periventricular diffusion marker related to interstitial fluid transport rather than a direct measurement of glymphatic fluid flow or function.

### White matter hyperintensity assessment

WMH burden was assessed separately in the left and right periventricular white matter and deep white matter using the available 0–3 semiquantitative regional ratings. The four regional ratings were summed to derive a total WMH burden score ranging from 0 to 12, with higher scores indicating greater WMH burden. Because these pre-existing regional ratings were used as a coarse structural burden measure rather than as a dedicated quantitative WMH biomarker, the summed score was included only as a sensitivity covariate to evaluate potential confounding by white-matter injury.

### Statistical analysis

Statistical analyses were performed using complete-case data. Continuous variables are presented as mean ± standard deviation or median with interquartile range, as appropriate, and categorical variables as counts and percentages. Variable distributions were assessed using histograms, Q-Q plots, and the Shapiro-Wilk test. Sex was coded as female = 0 and male = 1. Total WMH burden was summarized as median and interquartile range.

Associations between imaging markers and cognitive scores were examined using Spearman rank correlation. Correlations were calculated between MoCA/MMSE scores and left, right, and mean ALPS and PVeD indices. Age- and sex-adjusted associations were further assessed using partial Spearman correlation. To address multiple testing, the Benjamini-Hochberg false discovery rate (FDR) procedure was applied separately to the 12 unadjusted Spearman tests and the 12 age- and sex-adjusted partial Spearman tests. Nominal *P* values and FDR-adjusted q values are reported; *q* < 0.05 was considered statistically significant for these correlation analyses. For Supplementary Figure S1, approximate 95% confidence intervals for partial Spearman coefficients were calculated using Fisher z transformation with k = 2 covariates and were back-transformed to the correlation scale.

Multivariable linear regression was used to evaluate the independent associations of mean ALPS and mean PVeD with MoCA and MMSE scores. Mean ALPS and mean PVeD were entered in separate age- and sex-adjusted models to reduce collinearity and overfitting. To evaluate potential confounding by structural white-matter injury, the first sensitivity models additionally adjusted for total WMH burden. An extended sensitivity model further adjusted for maximum hemispheric Suzuki stage as a parsimonious patient-level measure of angiographic disease severity. Regression results are reported as unstandardized coefficients, 95% confidence intervals (CI), nominal *P* values, adjusted *R*-squared values, and overall model *P* values. Regression-model *P* values were considered exploratory and were not additionally adjusted for multiple testing. Model assumptions were checked using residual and influence diagnostics. All tests were two-sided.

For the laterality assessment, left and right Suzuki stage distributions were compared using the paired Wilcoxon signed-rank test to evaluate whether a systematic hemispheric difference in angiographic severity could account for the observed laterality pattern in the imaging-cognition correlations. Given the exploratory nature and modest sample size, no additional high-dimensional vascular covariate set was introduced.

## Results

### Participant characteristics

A total of 59 patients with asymptomatic moyamoya disease were included in the final analysis. The cohort included 34 women and 25 men, with a mean age of 44.22 ± 13.54 years. The mean MoCA and MMSE scores were 22.63 ± 5.29 and 26.12 ± 3.33, respectively. The mean ALPS index was 1.42 ± 0.18, and the mean PVeD index was 0.48 ± 0.04. The median total WMH burden score was 3 (interquartile range, 2–5; observed range, 0–9). Angiographically, 43/59 patients (72.9%) had bilateral involvement and 16/59 (27.1%) had unilateral involvement, including 10 with left-sided and 6 with right-sided involvement. The maximum hemispheric Suzuki stage was 1 in 8 patients (13.6%), 2 in 21 (35.6%), 3 in 20 (33.9%), and 4 in 10 (16.9%); the median maximum stage was 3 (interquartile range, 2–3) (Supplementary Table 1).

The median left and right Suzuki stages were both 2 (left interquartile range, 1.5–3; right interquartile range, 1–3), and their paired distributions did not differ significantly (Wilcoxon signed-rank *P* = 0.095). Thus, although unilateral left-sided involvement was somewhat more frequent than unilateral right-sided involvement (10 vs. 6 patients), there was no clear cohort-level leftward predominance in angiographic severity.

### Unadjusted correlations between ALPS, PVeD, and cognitive performance

In the unadjusted Spearman correlation analysis, the mean ALPS index was positively correlated with MoCA score (rho = 0.493, *P* < 0.001, *q* < 0.001) and MMSE score (rho = 0.326, *P* = 0.0118, *q* = 0.0272). Among the lateralized ALPS measures, the left ALPS index correlated with both MoCA (rho = 0.522, *P* < 0.001, *q* < 0.001) and MMSE (rho = 0.372, *P* = 0.0037, *q* = 0.0149), whereas the right ALPS index correlated with MoCA (rho = 0.335, *P* = 0.0095, *q* = 0.0272) but not MMSE (rho = 0.194, *P* = 0.1407, *q* = 0.1688) (Table 1).

**Table 1:** Spearman correlations of ALPS and PVeD with cognitive scores.

Imaging variable	Cognitive variable	Spearman rho	*P* value	FDR-adjusted *q*
ALPS index, mean	MoCA score	0.493	0.0001	0.0004^***^
ALPS index, left	MoCA score	0.522	< 0.001	0.0003^***^
ALPS index, right	MoCA score	0.335	0.0095	0.0272^*^
PVeD index, mean	MoCA score	0.246	0.0604	0.0805
PVeD index, left	MoCA score	0.282	0.0307	0.0526
PVeD index, right	MoCA score	0.186	0.1584	0.1728
ALPS index, mean	MMSE score	0.326	0.0118	0.0272^*^
ALPS index, left	MMSE score	0.372	0.0037	0.0149^*^
ALPS index, right	MMSE score	0.194	0.1407	0.1688
PVeD index, mean	MMSE score	0.257	0.0496	0.0743
PVeD index, left	MMSE score	0.320	0.0136	0.0272^*^
PVeD index, right	MMSE score	0.157	0.2351	0.2351

Values are presented as Spearman rho coefficients, nominal *P* values, and Benjamini-Hochberg FDR-adjusted *q* values. Significance markers are based on *q* values: ^*^q < 0.05; ^**^q < 0.01; ^***^q < 0.001.

For PVeD, the unadjusted associations were less consistent. The mean PVeD association with MMSE was nominally significant (rho = 0.257, *P* = 0.0496) but did not survive FDR correction (*q* = 0.0743), and mean PVeD was not associated with MoCA (rho = 0.246, *P* = 0.0604, *q* = 0.0805). Left PVeD correlated with MMSE after FDR correction (rho = 0.320, *P* = 0.0136, *q* = 0.0272), whereas its association with MoCA was borderline after correction (rho = 0.282, *P* = 0.0307, *q* = 0.0526). Right PVeD was not associated with either cognitive measure (Table 1).

The scatterplots in Figure 1 showed a similar pattern for the mean indices: mean ALPS showed clearer positive trends with MoCA and MMSE scores, whereas the associations for mean PVeD were more modest.

**Figure 1:**
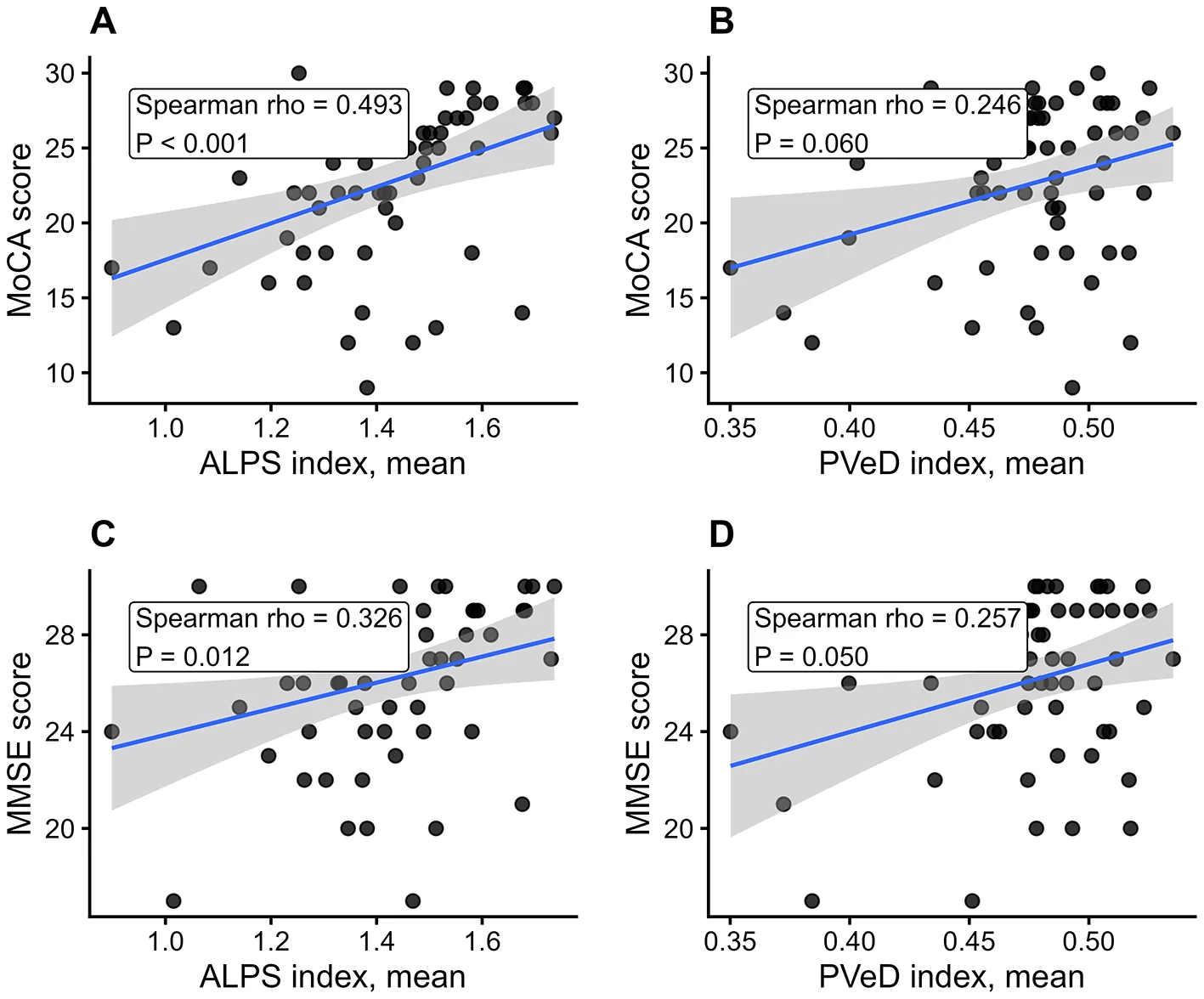
Associations of mean ALPS and PVeD with global cognitive performance in asymptomatic moyamoya disease. **(A)** Mean ALPS index versus MoCA score. **(B)** Mean PVeD index versus MoCA score. **(C)** Mean ALPS index versus MMSE score. **(D)** Mean PVeD index versus MMSE score. Scatter plots show the unadjusted relationships between imaging indices and cognitive scores. Solid lines indicate fitted linear trends, and shaded areas represent 95% confidence intervals. Spearman rank correlation coefficients and nominal *P* values are displayed within each panel; corresponding FDR-adjusted q values are reported in Table 1. ALPS, diffusion tensor image analysis along the perivascular space; PVeD, periventricular diffusivity; MoCA, Montreal Cognitive Assessment; MMSE, Mini-Mental State Examination.

### Age- and sex-adjusted partial correlations

After adjustment for age and sex, the mean ALPS index remained correlated with both MoCA (partial rho = 0.494, *P* < 0.001, *q* < 0.001) and MMSE (partial rho = 0.316, *P* = 0.0167, *q* = 0.0401). Similar results were observed for the left ALPS index, which correlated with MoCA (partial rho = 0.508, *P* < 0.001, *q* < 0.001) and MMSE (partial rho = 0.346, *P* = 0.0085, *q* = 0.0254). The right ALPS index remained associated with MoCA (partial rho = 0.349, *P* = 0.0078, *q* = 0.0254), but not with MMSE (partial rho = 0.203, *P* = 0.1300, *q* = 0.2229) (Table 2).

**Table 2:** Partial Spearman correlations adjusted for age and sex.

Imaging variable	Cognitive variable	Partial Spearman rho	*P* value	FDR-adjusted *q*
ALPS index, mean	MoCA score	0.494	< 0.001	0.0006^***^
ALPS index, left	MoCA score	0.508	< 0.001	0.0006^***^
ALPS index, right	MoCA score	0.349	0.0078	0.0254^*^
PVeD index, mean	MoCA score	0.147	0.2738	0.3285
PVeD index, left	MoCA score	0.180	0.1797	0.2396
PVeD index, right	MoCA score	0.099	0.4622	0.4927
ALPS index, mean	MMSE score	0.316	0.0167	0.0401^*^
ALPS index, left	MMSE score	0.346	0.0085	0.0254^*^
ALPS index, right	MMSE score	0.203	0.1300	0.2229
PVeD index, mean	MMSE score	0.182	0.1744	0.2396
PVeD index, left	MMSE score	0.243	0.0683	0.1365
PVeD index, right	MMSE score	0.093	0.4927	0.4927

Values are presented as partial Spearman rho coefficients, nominal *P* values, and Benjamini-Hochberg FDR-adjusted *q* values. Significance markers are based on q values: ^*^q < 0.05; ^**^q < 0.01; ^***^q < 0.001.

For PVeD, none of the age- and sex-adjusted correlations with MoCA or MMSE remained significant after FDR correction. The mean PVeD index was not correlated with MoCA (partial rho = 0.147, *P* = 0.2738, *q* = 0.3285) or MMSE (partial rho = 0.182, *P* = 0.1744, *q* = 0.2396). The association between left PVeD and MMSE approached nominal significance (partial rho = 0.243, *P* = 0.0683) but remained non-significant after FDR correction (*q* = 0.1365) (Table 2).

### Multivariable linear regression and WMH sensitivity analyses

In multivariable linear regression models adjusted for age and sex, a higher mean ALPS index was associated with higher MoCA (beta = 11.716, 95% CI: 4.930 to 18.502, *P* = 0.0011; adjusted *R*-squared = 0.233) and MMSE scores (beta = 5.213, 95% CI: 0.619 to 9.807, *P* = 0.0269; adjusted *R*-squared = 0.113). After additional adjustment for total WMH burden, the association between mean ALPS and MoCA remained significant (beta = 9.281, 95% CI: 2.512 to 16.051, *P* = 0.0081; adjusted *R*-squared = 0.301), whereas the association with MMSE was attenuated and no longer statistically significant (beta = 3.739, 95% CI: −0.897 to 8.375, *P* = 0.1117; adjusted R-squared = 0.172) (Table 3).

**Table 3:** Multivariable linear regression models.

Outcome	Predictor	Beta	95% CI	*P* value	Adjusted *R*-squared	Model *P* value	Adjustment
MoCA score	ALPS index, mean	11.716	4.930 to 18.502	0.0011	0.233	< 0.001	Age + sex
MoCA score	PVeD index, mean	34.662	−1.697 to 71.022	0.0613	0.125	0.0159	Age + sex
MMSE score	ALPS index, mean	5.213	0.619 to 9.807	0.0269	0.113	0.0225	Age + sex
MMSE score	PVeD index, mean	23.944	0.735 to 47.153	0.0434	0.099	0.0329	Age + sex
MoCA score	ALPS index, mean	9.281	2.512 to 16.051	0.0081	0.301	< 0.001	Age + sex + WMH
MoCA score	PVeD index, mean	21.208	−14.356 to 56.772	0.2371	0.223	0.0013	Age + sex + WMH
MMSE score	ALPS index, mean	3.739	−0.897 to 8.375	0.1117	0.172	0.0065	Age + sex + WMH
MMSE score	PVeD index, mean	16.813	−6.404 to 40.030	0.1523	0.164	0.0080	Age + sex + WMH
MoCA score	ALPS index, mean	7.790	0.816 to 14.765	0.0293	0.317	< 0.001	Age + sex + WMH + max hemispheric Suzuki
MoCA score	PVeD index, mean	11.216	−25.167 to 47.599	0.5390	0.258	< 0.001	Age + sex + WMH + max hemispheric Suzuki
MMSE score	ALPS index, mean	3.121	−1.721 to 7.963	0.2017	0.169	0.0104	Age + sex + WMH + max hemispheric Suzuki
MMSE score	PVeD index, mean	13.477	−10.850 to 37.804	0.2715	0.162	0.0125	Age + sex + WMH + max hemispheric Suzuki

Primary models were adjusted for age and sex; first sensitivity models were additionally adjusted for total WMH burden; extended sensitivity models additionally included maximum hemispheric Suzuki stage. Values are presented as unstandardized regression coefficients, 95% confidence intervals, nominal predictor *P* values, adjusted *R*-squared values, and overall model *P* values. Separate models were constructed for each cognitive outcome and each mean imaging marker. Overall model *P* values refer to the regression-model F test and should not be interpreted as the significance of the imaging predictor. Regression *P* values were exploratory and were not additionally adjusted for multiple testing.

For PVeD, the age- and sex-adjusted association with MoCA did not reach statistical significance (beta = 34.662, 95% CI: −1.697 to 71.022, *P* = 0.0613; adjusted *R*-squared = 0.125), whereas mean PVeD showed a nominal association with MMSE (beta = 23.944, 95% CI: 0.735 to 47.153, *P* = 0.0434; adjusted *R*-squared = 0.099). After additional adjustment for total WMH burden, mean PVeD was not associated with either MoCA (beta = 21.208, 95% CI: −14.356 to 56.772, *P* = 0.2371; adjusted *R*-squared = 0.223) or MMSE (beta = 16.813, 95% CI: −6.404 to 40.030, *P* = 0.1523; adjusted *R*-squared = 0.164) (Table 3).

After further adjustment for maximum hemispheric Suzuki stage in addition to age, sex, and total WMH burden, mean ALPS showed a persisting association with MoCA (beta = 7.790, 95% CI: 0.816 to 14.765, *P* = 0.0293; adjusted *R*-squared = 0.317), whereas its association with MMSE remained non-significant (beta = 3.121, 95% CI:−1.721 to 7.963, *P* = 0.2017; adjusted *R*-squared = 0.169). Mean PVeD was not associated with either MoCA (beta = 11.216, 95% CI: −25.167 to 47.599, *P* = 0.5390; adjusted *R*-squared = 0.258) or MMSE (beta = 13.477, 95% CI: −10.850 to 37.804, *P* = 0.2715; adjusted *R*-squared = 0.162) in the extended sensitivity models (Table 3).

## Discussion

The main finding of this Brief Research Report is that DTI-ALPS showed more consistent cross-sectional associations with global cognitive performance than PVeD in asymptomatic moyamoya disease. Chen et al. recently reported that periventricular diffusivity was associated with amyloid burden, neurodegeneration, cognitive impairment, and APOE epsilon4-related vulnerability across the AD continuum (6). After adjustment for age and sex and FDR correction, *five* of the *six* ALPS-cognition partial correlations remained significant: mean, left, and right ALPS with MoCA, and mean and left ALPS with MMSE. In contrast, *non*e of the *six* PVeD-cognition partial correlations remained significant after FDR correction. In sensitivity analyses, mean ALPS showed a persisting association with MoCA after adjustment for WMH burden and further adjustment for maximum hemispheric Suzuki stage, whereas the ALPS-MMSE association was attenuated; mean PVeD was not associated with either score in the extended sensitivity models. These findings do not contradict observations in AD cohorts but support cautious, disease-contextual interpretation of diffusion-based markers. Because the present study is cross-sectional, the observed differences represent concurrent imaging-cognition associations and should not be interpreted as evidence of causal effects or longitudinal predictive value.

The apparent left-sided predominance of the imaging-cognition associations also warrants cautious interpretation. Left-sided ALPS and PVeD measures generally showed stronger correlations with global cognitive scores than their right-sided counterparts; however, bilateral Suzuki staging did not demonstrate a significant left-right difference in angiographic severity (*P* = 0.095). Therefore, a systematic excess of left-sided angiographic disease burden does not readily explain the observed correlation pattern. Other possibilities include hemispheric functional lateralization, local differences in white-matter architecture, measurement variability, or chance in the setting of multiple exploratory comparisons. Because MoCA and MMSE include language-dependent components, left-hemisphere functional dominance could contribute to the pattern, but the present study was not designed to test this mechanism. Moreover, we did not formally test whether the left- and right-sided correlation coefficients differed from one another; the apparent laterality should therefore be regarded as exploratory rather than as evidence of a true hemispheric difference.

A plausible explanation is that periventricular diffusivity reflects different tissue processes in AD and chronic cerebral ischemia. In the AD continuum, PVeD may be related to amyloid deposition, APOE-related susceptibility, and neurodegenerative changes (6). In moyamoya disease, the dominant pathophysiological background is different. Progressive intracranial arterial stenosis, long-standing hypoperfusion, vascular remodeling, impaired hemodynamic reserve, blood-brain barrier dysfunction, and ischemia-related white matter injury may all contribute to cognitive impairment (7, 10). These vascular and ischemic mechanisms can alter water diffusion in periventricular white matter through pathways that are not specific to glymphatic or interstitial fluid clearance.

This issue is particularly relevant for PVeD because the periventricular region is vulnerable to structural confounders. White matter hyperintensities, demyelination, tissue rarefaction, ventricular enlargement, age-related atrophy, and partial-volume effects may all influence diffusion measurements around the lateral ventricles (11, 12). In the present study, total WMH burden was therefore incorporated into sensitivity models. Additional adjustment for WMH attenuated the nominal PVeD-MMSE association and the ALPS-MMSE association, whereas the ALPS-MoCA association persisted. Further adjustment for maximum hemispheric Suzuki stage did not remove the ALPS-MoCA association, although the effect estimate was attenuated. This pattern suggests that structural white-matter injury and angiographic disease severity account for part, but not necessarily all, of the observed imaging-cognition relationships. In chronic hypoperfusion, PVeD may therefore represent a composite signal reflecting periventricular microstructural injury, ventricular morphology, tissue water content, ischemia-related diffusion changes, and potentially interstitial fluid transport, rather than a specific measure of glymphatic function.

Our findings also emphasize that DTI-ALPS and PVeD should not be regarded as interchangeable measures of the same biological process. DTI-ALPS was designed to estimate directional diffusivity along medullary perivascular spaces by using diffusion information from projection and association fibers (3, 5). PVeD, in contrast, measures diffusivity in ventricular-adjacent tissue. The two metrics therefore differ in anatomical definition, diffusion directionality, region-of-interest dependence, and susceptibility to local tissue architecture. Their relationships with cognition may accordingly vary across diseases, depending on whether the dominant pathology involves amyloid-related neurodegeneration, vascular hypoperfusion, periventricular white matter injury, or a combination of these processes.

It is also important to note that neither DTI-ALPS nor PVeD directly measures glymphatic flow. DTI-ALPS has been widely used as a non-invasive imaging marker related to glymphatic function, but it is affected by white matter microstructure, diffusion geometry, region-of-interest placement, scanner parameters, and disease-related tissue changes (5, 13, 14). Nevertheless, DTI-ALPS has undergone broader methodological evaluation than PVeD, including studies of its diffusion-anatomical rationale, reproducibility, and relationship with intrathecal contrast-enhanced MRI markers of human CSF tracer distribution and clearance (5, 15–17). These studies do not establish DTI-ALPS as a direct measure of glymphatic function, and recent evidence suggests that its agreement with tracer-based MRI markers may be limited (17). However, they provide a more developed methodological framework for interpreting DTI-ALPS than is currently available for PVeD.

The present findings should therefore be interpreted cautiously. They do not show that DTI-ALPS is a superior marker of true glymphatic function. Rather, under the present measurement conditions, DTI-ALPS was more closely and consistently associated with global cognitive performance than PVeD in asymptomatic moyamoya disease. Before PVeD is applied as a general marker of glymphatic dysfunction across disease populations, further work is needed to clarify what biological processes it captures, how it relates to tracer-based reference methods, and how it is affected by ventricular morphology, periventricular white matter injury, aging, and vascular disease.

Several limitations should be considered. First, this was a cross-sectional study with a relatively small sample size. Therefore, the directionality or causality of the observed imaging-cognition associations cannot be determined, and the ability of DTI-ALPS or PVeD to predict subsequent cognitive decline could not be assessed. Second, APOE genotype and amyloid-beta or tau biomarkers were unavailable, so occult AD pathology, mixed vascular-neurodegenerative pathology, or APOE-related effects could not be excluded. Education level was also unavailable and may have influenced MoCA and MMSE performance. Third, although WMH burden and maximum hemispheric Suzuki stage were incorporated into sensitivity analyses, other potential structural and hemodynamic confounders, including ventricular volume, brain atrophy, lacunes, cerebral microbleeds, quantitative cerebral perfusion, and cerebrovascular reserve, were not quantitatively modeled, and residual confounding remains possible. Fourth, cognition was assessed using MoCA and MMSE, which are global screening instruments rather than a comprehensive neuropsychological battery. Accordingly, the absence of a PVeD association with global scores does not exclude relationships with specific domains such as executive function, processing speed, or attention.

Finally, this study compared the cognitive associations of DTI-ALPS and PVeD but could not determine which metric better reflects true glymphatic function. DTI-ALPS remains an indirect diffusion-based proxy, and PVeD requires further validation against tracer-based reference methods and across vascular and neurodegenerative populations (13, 17). Future prospective studies should integrate longitudinal cognitive follow-up, quantitative cerebral perfusion and cerebrovascular reserve measurements, structural vascular brain injury markers, AD biomarkers, and reference approaches to glymphatic assessment where feasible. Comprehensive neuropsychological batteries emphasizing executive function, processing speed, attention, and memory would also help clarify domain-specific imaging-cognition relationships in vascular cognitive impairment.

Taken together, these cross-sectional results support a disease-specific, structurally informed, and angiographically contextualized interpretation of diffusion-based markers of brain fluid transport. PVeD may be informative in amyloid-dominant AD cohorts, as reported by Chen et al. (6), but its association with global cognitive performance appears less consistent in this chronic hypoperfusion cohort and is sensitive to structural white-matter injury. DTI-ALPS showed more consistent associations with global cognition, particularly for MoCA, and this association persisted after additional adjustment for WMH burden and maximum hemispheric Suzuki stage; however, DTI-ALPS should still be interpreted as an indirect diffusion-based marker rather than a direct measure of glymphatic clearance. Longitudinal and multimodal studies are needed before either metric can be considered predictive of cognitive trajectories.

## Data Availability

The data that support the findings of this study are available from the corresponding author upon reasonable request, subject to institutional regulations and protection of patient privacy.

## Supplementary material

Supplementary Figure S1 Age- and sex-adjusted partial Spearman correlations between ALPS/PVeD measures and global cognitive scores. Points indicate partial Spearman rho coefficients and horizontal bars indicate approximate 95% confidence intervals calculated using Fisher z transformation and back-transformed to the correlation scale. FDR-adjusted q values are displayed in a separate right-hand column to avoid overlap with confidence intervals. This figure complements Table 2 by providing a direct visual summary of covariate-adjusted associations.
