# Supplementary material for: Disease-context dependence of periventricular diffusivity and DTI-ALPS in asymptomatic moyamoya disease
Source: Front Neurol. 2026 Oct 21;17:1927018. doi: 10.3389/fneur.2026.1927018 (PMC13730843; doi:10.3389/fneur.2026.1927018)
Supplement: Supplementary file 1 [file Table_1.docx]

**Supplementary Table 1. Baseline characteristics of the study cohort**

| **Variable** | **N** | **Mean ± SD / n (%)** | **Median [IQR]** | **Range** |
| --- | --- | --- | --- | --- |
| Number of complete cases | 59 | — | — | — |
| Female sex | 59 | 34 (57.6%) | — | — |
| Male sex | 59 | 25 (42.4%) | — | — |
| Age, years | 59 | 44.22 ± 13.54 | 45.00 [35.00, 53.50] | 18.00–68.00 |
| MoCA score | 59 | 22.63 ± 5.29 | 24.00 [18.50, 27.00] | 9.00–30.00 |
| MMSE score | 59 | 26.12 ± 3.33 | 27.00 [24.00, 29.00] | 17.00–30.00 |
| Total WMH burden score | 59 | — | 3 [2, 5] | 0–9 |
| Bilateral angiographic involvement | 59 | 43 (72.9%) | — | — |
| Unilateral angiographic involvement | 59 | 16 (27.1%) | — | — |
| Left-sided unilateral involvement | 59 | 10 (16.9%) | — | — |
| Right-sided unilateral involvement | 59 | 6 (10.2%) | — | — |
| Left hemispheric Suzuki stage | 59 | — | 2 [1.5, 3] | — |
| Right hemispheric Suzuki stage | 59 | — | 2 [1, 3] | — |
| Maximum hemispheric Suzuki stage | 59 | — | 3 [2, 3] | 1–4 |
| Maximum hemispheric Suzuki stage 1 | 59 | 8 (13.6%) | — | — |
| Maximum hemispheric Suzuki stage 2 | 59 | 21 (35.6%) | — | — |
| Maximum hemispheric Suzuki stage 3 | 59 | 20 (33.9%) | — | — |
| Maximum hemispheric Suzuki stage 4 | 59 | 10 (16.9%) | — | — |
| ALPS index, mean | 59 | 1.42 ± 0.18 | 1.42 [1.31, 1.53] | 0.90–1.74 |
| ALPS index, left | 59 | 1.43 ± 0.21 | 1.45 [1.33, 1.58] | 0.76–1.85 |
| ALPS index, right | 59 | 1.40 ± 0.20 | 1.41 [1.26, 1.55] | 0.84–1.81 |
| PVeD index, mean | 59 | 0.48 ± 0.04 | 0.48 [0.46, 0.50] | 0.35–0.54 |
| PVeD index, left | 59 | 0.47 ± 0.04 | 0.47 [0.46, 0.49] | 0.34–0.53 |
| PVeD index, right | 59 | 0.48 ± 0.04 | 0.49 [0.47, 0.51] | 0.34–0.55 |

Values are presented as mean ± standard deviation, median [interquartile range], range, or n (%), as appropriate. WMH burden was calculated by summing the available left/right periventricular and left/right deep white matter hyperintensity ratings (each scored 0–3), yielding a total score of 0–12; the observed range in this cohort was 0–9. Suzuki stages were recorded separately for each hemisphere. For analytic purposes, an angiographically uninvolved hemisphere was coded as 0; this coding does not represent a conventional Suzuki stage. The maximum hemispheric Suzuki stage was used as the patient-level angiographic severity covariate in sensitivity analyses. ALPS, diffusion tensor image analysis along the perivascular space; PVeD, periventricular diffusivity; WMH, white matter hyperintensity; MoCA, Montreal Cognitive Assessment; MMSE, Mini-Mental State Examination.
